# The "Win-Win" initiative: a global, scientifically based approach to resource sparing treatment for systemic breast cancer therapy

**DOI:** 10.1186/1477-7819-7-44

**Published:** 2009-05-05

**Authors:** Ahmed Elzawawy

**Affiliations:** 1 Clinical Oncology Department, Faculty of Medicine, Suez Canal University, Egypt; 2 Alsoliman Radiation Oncology Unit, Port Said, Egypt; 3 Early Detection and Cancer Chemotherapy Unit, Port Said General Hospital, Egypt; 4 ICEDOC: International Campaign for Establishment and Development of Oncology Centers & ICEDOC's Experts in Cancer without Borders, USA; 5 SEMCO: South and East Mediterranean college of Oncology, Egypt

## Abstract

**Background:**

Worldwide, breast cancer is the most frequent malignancy among females. Its incidence shows a trend towards an increase in the next decade, particularly in developing countries where less than of 5% of resources for cancer management are available. In most breast cancer cases systemic cancer treatment remains a primary management strategy. With the increasing costs of novel drugs, amidst the growing breast cancer rate, it can be safely assumed that in the next decade, newly developed cancer drugs will become less affordable and therefore will be available to fewer patients in low and middle income countries. In light of this potentially tragic situation, a pressing need emerges for science-based innovative solutions.

**Methods:**

In this article, we cite examples of recently published researches and case management approaches that have been shown to lower overall treatment costs without compromising patient outcomes. The cited approaches are not presented as wholly inclusive or definitive solutions but are offered as effective examples that we hope will inspire the development of additional evidence-based management approaches that provide both efficient and effective breast cancer treatment

**Results:**

We propose a "win-win" initiative, borne in the year of 2008 of strategic information sharing through preparatory communications, publications and our conference presentations. In the year 2009, ideas developed through these mechanisms can be refined through focused small pilot meetings with interested stakeholders, including the clinical, patient advocate, and pharmaceutical communities, and as appropriate (as proposed plans emerge), governmental representatives. The objective is to draw a realistic road map for feasible and innovative scientific strategies and collaborative actions that could lead to resource sparing; i.e. cost effective and tailored breast cancer systemic treatment for low and middle income countries.

**Conclusion:**

The intended result would assure sustained affordability and accessibility in breast cancer systemic therapy for patients in low and middle income countries. As an added benefit, the example of breast cancer could be expanded to include other cancers in diverse settings around the world.

## Background

By the year 2020, 70% of the twenty million new cancer cases will occur in countries that collectively have only five percent of the global resources for cancer control [1]. Breast cancer is the most frequent cancer among females. Globally the incidence of breast cancer is increasing, and the rate of increase is highest in developing countries. [2].

This trend provides every indication that the need for systemic anticancer agents will continue to increase over the next ten years. The pharmaceutical companies are developing increasingly expensive novel anticancer molecules with no indication that the rapidly escalating cost of new treatments will ease in future. Improvements in the overall and disease-free survival rates and quality of life are not commensurate with the soaring costs of cancer treatment.

The major markets for the leading pharmaceutical industry are in the United States, Western Europe and Japan; and while these regions may be able to meet the increased cost of treatment, it can be safely assumed that the cost of novel anticancer drugs will continue to expand as an insurmountable obstacle to care for an ever greater proportion of cancer patients in Low and Middle Income Countries (LMCs) where the majority of the world's population live. This discomforting reality confronts us with difficult challenges that merit the spirited engagement of regional and international health leaders.

Breast Cancer, with its predictable increase in incidence, and multiple available, effective treatment options provides an excellent starting point for developing economically sustainable cancer control strategies that could be tailored in LMCs for other forms of cancer as well.

### Aims and hopes

It is our aim to establish a scientific initiative to expand availability of resource sparing Breast Cancer Systemic Therapy (BCST) and hope that such strategy may meet the demand for effective, affordable breast cancer care for patients who would otherwise be left without scientifically valid treatment options.

## Methods

This communication reviews examples of recent and ongoing scientific researches and suggestions that could lead to lower costs of BCST without compromising overall patient outcomes. These findings, this summary, and subsequent detailed publications, and conference presentations can provide a basis for pilot meetings to launch a "win-win" scientific initiative based on cooperation and collaboration of stakeholders; whereby markets are created or maintained for effective cancer therapies, and patients are assured access to these interventions regardless of where in the world they reside.

## Results

### A) Relatively recent drugs

#### The duration of the course trastuzumab (Herceptin^®^)

A trial of 9-weeks of trastuzumab treatment has been compared to 52 weeks treatment. Both arms were similar in outcome [3]. We assume that these preliminary results need to be confirmed in a larger sample. In addition to the cost savings from the shortened treatment interval with trastuzumab, we could expect further reduction in costs due to fewer hospitalizations and less need for supportive treatment.

#### Evidence based cost effective prescription of drugs

Limiting use of *trastuzumab (Herceptin^®^) *to women with localized disease and known HER2/neu-positive status, as suggested by Yarney and colleagues[4] is a cost-effective use if resources are available, even with the additional costs of HER2/new testing.

#### Low dose, prolonged infusion gemcitabine

The encouraging response of phase I-II trials of low dose gemcitabine in prolonged infusion in the treatment of certain solid cancers, e.g. non small cell lung cancer, breast, pancreas and bladder cancers deserves further investigation. The explanation for these responses caused by low doses (of 250 mg and 180 mg/m^2 ^for 6, 24 hours respectively) lies in the saturation of deoxcytidine kinase which occurs after short infusion at conventional doses. This enzyme is needed for conversion of gemcitabine into its active form gemcitabine triphosphate. While short usual infusion leaves most of the drug unmetabolized, prolonged infusion apparently leads to a higher intracellular concentration of the active metabolite [5]. More studies are needed in different clinical settings to verify the effectiveness and cost implications of extended (six hours infusion), using reduced drug dosages.

#### The Glivec^® ^International Patient Assistance Program (GIPAP)

is a worldwide program to provide imatinib (Glivec^®^) at no cost to patients with chronic myelogenous leukemia (CML) or gastrointestinal stromal tumor (GIST) in 81 countries who would not otherwise have access to comprehensive reimbursement for the treatment[6]. This example could be explored for other drugs with other parties.

#### Interrupted courses of treatment

Aromatase inhibitors (AI), when given as interrupted course, probably would also be effective as continuous therapy after prior tamoxifen and/or AI treatment. The hypothesis is that AI interrupted courses may cause estrogenic stimulation and enhance response of residual resistant cells[7]. We cite this example not for application as a finally proven scientific and economically sound approach, but as an example of the possibilities for interrupting the continuity of a treatment course without compromising the result. The principle implied by this intriguing example merits further study.

#### Pharmacokintetic based studies in lapatinib therapy

According to recent studies, it is shown that lapatinib when taken orally with food -not on an empty stomach as cited frequently–yields an increased plasma level. Lower oral doses administrated with food and with grapefruit juice could effectively inhibit the enzyme CYP3A and thereby could provide comparable effect. Up to 80% of the dose and the cost of the drug could be reduced by this approach [8]. More studies are needed in this field.

**- There are greater possibilities in using oral cancer drugs **[9]. The oral route for cancer therapy may decrease costs due to fewer hospital inpatient admissions and outpatient chemotherapy intravenous sessions. Obviously, this approach requires careful study in diverse communities. Questions of cost-effectiveness and best practices relating to oral and self-administered agents are of considerable interest in LMCs where facilities and providers may be particularly scarce.

### B) Essential and conventional systemic cancer drugs

The following concise points relate to what some refer to as "the essential and conventional" systemic cancer drugs:

Fortunately, the pharmaceutical arsenal of "essential and conventional systemic anticancer drugs" still constitutes the basis of this treatment modality. In addition, these conventional drugs are relatively inexpensive. For breast cancer the list would include CMF (Cyclophosphamide, Methotrexate and 5 Fluorouracil), FAC (5 Fluorouracil, Doxorubicin and Cyclophosphamide), Tamoxifen and Ovarian ablation.

Innovative strategic thinking and approaches should be encouraged to improve the availability and accessibility of first-line systemic anticancer treatments as part of the comprehensive breast cancer control plan for underserved countries. An example of novel chronology and mode of drug administration that tests additional mechanisms of actions and indications is the metronomic use of prolonged, low oral doses of cyclophosphamide and methotrexate as palliative breast cancer treatment [10]. While an example of new applications for relatively old and less expensive drug is the use of Cisplatin in triple negative breast cancer patients [11].

#### Generic equivalents for off-patent drugs

offer the possibility of less expensive treatment. However, the quality and bioequivalence of generics used in developing countries should be assured by regulations or developing a transparent system for international testing. To overcome difficulties in achieving large scale feasibility in quality control, we suggest working at the small scale level to test random samples or pilot settings upon invitation from the local authorities in some developing countries.

#### Pharmcogenomic studies

Tamoxifen requires enzymatic activation by CYP 450 enzymes for the formation of clinically relevant metabolites 4-OH-tamoxifen and endoxifen which both have a greater affinity to the estrogen receptors and ability to inhibit cell proliferation when compared to the parent drug. The key enzyme in this bio-transformation is the CYP2D6. Recent pharmacological and clinical pharmacogenetics evidence suggests that genetic variants and drug interaction by CYP2D6 inhibitors influence plasma concentration of active tamoxifen metabolites and thereby improve treatment outcome of breast cancer patients treated by adjuvant tamoxifen [12]. It is speculated that the benefit of 5 years of adjuvant tamoxifen may even exceed that achieved through "upfront" AI treatment in postmenopausal CYP2D6 wt/wt genotype patients. Despite its expenses and technological requirements, we assume that, CYP2D6 genotyping prior to treatment could open new avenues for individualization of endocrine treatment. Hence, unnecessary treatment and costs extending over years could be avoided. However, we stress the need for wider studies to test cost effectiveness in different communities and the feasibility of technology transfer via international scientific cooperation.

Another approach would be to help develop the required infrastructure for the clinical research and trials with pragmatic goals that would be ideally suited to LMCs, few suggestions for this are:

##### For older drugs

a) To tailor treatment to patients, community and tumor factors (based on clinical, pathological and biological factors).

b) To address economic considerations, cost-effectiveness and quality of life issues within each community or region.

c) To test innovative combinations or different schedules of administration of older (and relatively cheaper) drugs that might lead to previously improved therapeutic index or applicability to specific societies. Such investigations usually are not supported by pharmaceuticals companies and international conferences, although they might be of benefit to science and cancer patients in LMCs.

d) To test new uses of a relatively old and less expensive drugs (as earlier cited in the use of Cisplatin in triple negative breast cancer patients).

##### For novel drugs

e) Conducted with appropriate ethical guidelines and international oversight, clinical research would provide broader and more transparent access to novel drugs, and would enhance professional education and training in LMCs. Such an approach would assist companies in streamlining the development of new drugs.

## Future directions and conclusion

On behalf of ICEDOC's Experts in Cancer without borders (ICEDOC is The International Campaign for Establishment and Development of Oncology Centers ), we propose "The win-win initiative". It starts with communications and publications. Then, we propose to hold small pilot working meetings with representatives of interested parties and willing leading pharmaceutical companies. Next, we would look to larger meetings and collaborative actions with a broader range of participants.

The goals of this approach include:

• To create a Think Tank or what Franklin Roosevelt described as "A Brain Trust", opened for innovative scientific thoughts and ideas that could lead to cost -effective systemic treatment for more breast cancer patients in LMCS.

• To develop a strategy to coordinate ongoing efforts and initiatives.

• To recommend areas of innovative clinical research that address the needs of LMCs

• To identify key components and infrastructure needs in LMCs to support research

• To determine the key barriers to pharmaceutical companies in investing in LMCs research and provide a strategy to overcome those barriers.

-Investment in creating infrastructure will create new markets

-Old drugs can bring new profits and its use may pave ways for novel drugs.

The outcome of the communications and meeting for the year 2009 would be a road map for the win-win initiative

• An actionable strategy opened for contribution, collaboration and coordinating efforts and ideas.

• Formation of a collaborative task force group that aims at more availability and affordability of systemic cancer therapy in LMCs.

• A published report on recommended research and key components.

• A proposal of pilot projects.

Finally, no leadership role, or claim to invention is being made. There are many key players and stakeholders in the world. Coordination and cooperation are needed.

## Competing interests

The author declares that they have no competing interests.
